# Cost-effectiveness analysis of ivonescimab plus chemotherapy in advanced squamous NSCLC: a Chinese healthcare system perspective with U.S. pricing scenario exploration

**DOI:** 10.3389/fphar.2026.1942976

**Published:** 2026-10-27

**Authors:** Linxiong Zheng, Kailong Zhong, Fang Chen, Jiayong Yang

**Affiliations:** 1 The First Affiliated Hospital of Xiamen University, Xiamen, Fujian, China; 2 Department of Pharmacy, Zhongshan Hospital Fudan University Xiamen Branch, Xiamen, Fujian, China; 3 Clinical Research Center for Cancer Therapy, Zhongshan Hospital Fudan University, Xiamen, Fujian, China; 4 Xiamen Professional Quality Control Center for Comprehensive Pharmaceutical Affairs Management, Xiamen, Fujian, China

**Keywords:** chemotherapy, cost-effectiveness analysis, HARMONi-6 trial, ivonescimab, non-small cell lung cancer, tislelizumab

## Abstract

**Background:**

Ivonescimab plus chemotherapy shows outstanding efficacy for advanced squamous non-small cell lung cancer (NSCLC), but its pharmacoeconomic value remains to be determined. Therefore, this study seeks to evaluate the cost-effectiveness of the ivonescimab regimen for advanced squamous NSCLC from the Chinese and U.S. healthcare perspectives, and to explore the theoretical cost-effective price threshold of ivonescimab.

**Methods:**

Survival curves from the HARMONi-6 trial were fitted and extrapolated, and direct medical costs were calculated. A partitioned survival model was developed within TreeAge Pro to project disease trajectory and resource utilization over a 10-year time horizon. The primary outcome was the incremental cost-effectiveness ratio (ICER), compared against willingness-to-pay (WTP) to assess the ivonescimab regimen’s cost-effectiveness. Sensitivity, scenario, and threshold analyses were performed to assess parameter uncertainty and estimate ivonescimab’s theoretical cost-effective price threshold.

**Results:**

In China, the ivonescimab group cost $60,942 for 2.33 QALYs versus $30,093 for 1.57 QALYs in the tislelizumab group, yielding an ICER of $40,328/QALY. Utility during progressive disease was the most influential parameter. Ivonescimab’s theoretical cost-effective price thresholds were $27.85/100mg, $67.63/100mg, and $107.4/100mg, corresponding to WTPs of single, double, and triple the per-capita GDP, respectively. In the U.S., the total costs were $572,981 and $419,922, and the health outcomes were 3.16 QALYs and 2.19 QALYs for the two arms, with an ICER of $157,444/QALY. Ivonescimab’s price was the dominant driver of ICER. The theoretical cost-effective price thresholds were $609.10/100 mg and $777.31/100mg, corresponding to the $100,000/QALY and $150,000/QALY WTP, respectively.

**Conclusion:**

Under the unified model framework of this study, compared with the tislelizumab regimen, the ivonescimab regimen as the first-line treatment for advanced squamous NSCLC in both countries is unlikely to have a cost-effectiveness advantage.

## 1 Introduction

Driven by population aging, environmental pollution and tobacco smoking, lung cancer imposes an increasing disease burden. Lung cancer ranks as the commonest malignant tumor in China and is simultaneously the deadliest cancer in both China and the U.S (Bray et al., 2024; Wu et al., 2024). The latest epidemiological data from China reported 1,175,900 newly diagnosed lung cancer cases (22.8% of all incident cancers) and 743,300 lung cancer deaths (28.8% of total cancer deaths) in 2024 (Xia et al., 2026). The American Cancer Society projected 229,410 new lung cancer cases (10.85% of incident malignancies) and 124,990 lung cancer deaths (19.96% of cancer mortality) in the U.S. in 2026 (Siegel et al., 2026). Non-small cell lung cancer (NSCLC) is the predominant subtype, accounting for 80%–85% of all lung cancers (Chen et al., 2014; Chen et al., 2022). Owing to atypical early manifestations, most patients are diagnosed at advanced stages, with a 5-year survival rate below 20% (Siegel et al., 2020; Travis et al., 2015).

Over recent decades, the pharmacotherapy of NSCLC has undergone substantial evolution, shifting from chemotherapy-alone regimens to precision therapy centered on targeted agents, immunotherapy and antibody-drug conjugates. Programmed cell death-1 (PD-1)/programmed death-ligand 1 (PD-L1) inhibitors counteract tumor immune escape, reinvigorate T-cell anti-tumor immunity, and have consequently yielded marked survival benefits in affected patients (Topalian et al., 2016). Vascular endothelial growth factor (VEGF), however, drives pathological neovascularization, obstructs T-cell infiltration, and bolsters immune evasion through PD-L1 upregulation on tumor cells, thereby limiting the clinical benefits of single-target immunotherapeutic agents (Lee et al., 2020). Bispecific antibody (BsAb) target distinct epitopes simultaneously, enabling concurrent blockade of the PD-(L)1 and VEGF pathways to boost anti-tumour immunity (Sun et al., 2023).

Ivonescimab is the first BsAb targeting both PD-1 and VEGF globally (Dhillon, 2024). The HARMONi-6 trial compared ivonescimab coupled with chemotherapy with tislelizumab coupled with chemotherapy as frontline options for advanced squamous NSCLC (Chen et al., 2025; Lu et al., 2026). The findings revealed that participants in the ivonescimab arm exhibited substantially extended median progression-free survival (mPFS) and median overall survival (mOS) relative to those receiving tislelizumab (mPFS: 11.1 months vs. 6.9 months, HR: 0.6, mOS: 27.89 months vs. 23.69 months, HR: 0.66).

Although the efficacy of ivonescimab in advanced squamous NSCLC has been fully verified, its substantial research and production costs may translate into high drug pricing, imposing a heavy economic burden on patients and the health system. A previous study has shown that, from the perspective of the Chinese healthcare system, compared with chemotherapy, ivonescimab combined with chemotherapy in the second-line treatment of advanced non-squamous NSCLC patients with positive epidermal growth factor receptor (EGFR) mutations has no cost-effectiveness advantage (Long et al., 2025). However, the substantial heterogeneity in pricing structures and WTP levels across China and the United States renders regional variation in drug cost-effectiveness all but inevitable. Accordingly, this study aimed to evaluate the cost-effectiveness of first-line ivonescimab plus chemotherapy versus tislelizumab plus chemotherapy for advanced squamous NSCLC from Chinese and U.S. healthcare perspectives, and estimated theoretical cost-effective price threshold of ivonescimab, thereby providing economic evidence for health decision-making.

## 2 Methods

### 2.1 Study design

This study was formulated and executed rigorously in alignment with the Consolidated Health Economic Evaluation Reporting Standards checklist (Supplementary Table S1) (Husereau et al., 2022). Data for this research came from publicly available Phase III clinical trials (the HARMONi-6 trial), the Menet.com database (https://www.menet.com.cn/), the Drugs.com price guide (https://www.drugs.com/price-guide/), and published studies. Since no individual-level patient data were incorporated, institutional ethical review and informed consent were not required.

This research leveraged data from the HARMONi-6 study to develop a modeling framework. Therefore, the target population aligned with the participant cohort recruited in the HARMONi-6 study, comprising individuals aged ≥18 years with pathologically confirmed, surgically unresectable stage IIIB–IV squamous NSCLC who had not previously received systemic anticancer therapy (Lu et al., 2026). Eligible participants were randomly allocated to receive four cycles of either ivonescimab (20 mg/kg, q3w) or tislelizumab (200mg, q3w) plus paclitaxel (175 mg/m^2^, q3w) and carboplatin (AUC = 5, q3w), followed by sequential treatment with the same dose of ivonescimab or tislelizumab until disease progression. However, the duration of using ivonescimab or tislelizumab should not exceed 24 months, and the earlier event would prevail. After the disease progressed, the treatment shifted to second-line therapy. According to the HARMONi-6 trial, 36% of ivonescimab group patients and 37% of tislelizumab group patients received subsequent-line therapy. Following the recommendations of the Chinese Society of Clinical Oncology (CSCO) Clinical Practice Guidelines for NSCLC (version: 2026) and the National Comprehensive Cancer Network (NCCN) Clinical Practice Guidelines in Oncology: NSCLC (Version 4. 2026), docetaxel (75 mg/m^2^, q3w) was chosen as the subsequent-line treatment drug, and best supportive care was provided for patients who did not proceed to second-line treatment (Riely et al., 2026; CSCO Oncology, 2026). To accurately calculate the dosage of the drug, this study assumes that the weight of Chinese patients is 65 kg, the body surface area (BSA) is 1.72 m^2^, and the creatinine clearance rate (CCR) is 70 mL/min (Luo et al., 2022; Zhou W. et al., 2025). For American patients, the weight is 70 kg, the BSA is 1.79 m^2^, and the CCR is 73 mL/min (Luo et al., 2022; Zhou W. et al., 2025).

### 2.2 Model overview

A partitioned survival model was constructed via TreeAge Pro software (version 2022). The modeling framework was structured around three mutually exclusive health states: progression-free survival (PFS), progressive disease (PD), and death. PFS was defined as the starting state, and death was specified as the absorbing state. The model structure is schematized in Supplementary Figure S1. The dosing regimen informed our selection of a 3-week cycle. A 10-year time horizon was applied based on the extrapolation results of the Kaplan–Meier (K–M) curves from the HARMONi-6 trial and the actual survival situation of advanced NSCLC. The model output parameters included total cost, incremental cost, quality-adjusted life-years (QALYs), incremental QALYs, and incremental cost-effectiveness ratio (ICER).

### 2.3 Survival estimate

Firstly, the Engauge Digitizer software was applied to digitize the K-M survival curves from the HARMONi-6 trial. The extracted data were then used to reconstruct individual patient data for each K–M curve following the methodological approach described by Guyot P et al. (Guyot et al., 2012). A comparative analysis of the median survival times derived from the reconstructed curves versus those reported in the original clinical trial is presented in Supplementary Table S2. Secondly, the survHE package embedded within the R software was invoked to fit and reconstruct individual-level patient data using six distribution functions (Exponential, Gamma, Weibull, Log-logistic, Log-normal, and Gompertz) from the standard parameter survival model. Model fit to the observed survival data was evaluated based on the Akaike information criterion (AIC) and the Bayesian information criterion (BIC). Combined with the shape of the risk function and the opinions of clinical experts, a comprehensive judgment was made on the rationality of the extrapolation. Supplementary Table S3 details the AIC and BIC values for each distribution. Finally, the log-logistic distribution provided the best fit to the PFS curves of both groups and the OS curve of the ivonescimab group, whereas the log-normal distribution provided the best fit to the OS curve of the tislelizumab group. Supplementary Table S4 lists the fitted parameters for each best-fit model, and Supplementary Figure S2 plots the fitted survival curves.

### 2.4 Cost and utility estimate

The model only incorporates direct medical expenditures, comprising drug costs, management costs associated with serious adverse reactions (ADRs), routine follow-up charges, best supportive care costs, and terminal care costs. Chinese drug cost data came from the Menet database, reflecting the winning bid prices in public hospital centralized procurement. U.S. drug cost data came from the Drug Price Guide, reflecting retail market prices in the U.S. Ivonescimab has not yet been approved for marketing in the U.S., so its price was estimated based on its domestic selling price. The cost of managing severe ADRs considered only grade 3 or higher ADRs with an incidence rate of ≥5%, and was calculated only once. Other medical costs came from previously published studies, and costs not in 2026 were converted to 2026 values using a discount rate. All costs were expressed in U.S. dollars, and the average exchange rate of CNY against the U.S. dollar in 2025 was used (1 USD ≈7.143 CNY), as detailed in Table 1.

**TABLE 1:** Model parameters.

Variable	Baseline value	Range	Distribution	Source
Cost (US$): China				
Ivonescimab per 100 mg	103.04	82.43∼123.65	Gamma	*
Tislelizumab per 100 mg	175.49	140.39∼210.59	Gamma	*
Paclitaxel per 30 mg	8.58	1.94∼68.5	Gamma	*
Carboplatin per 100 mg	5.78	4.62∼6.93	Gamma	*
Docetaxel per 20 mg	5.61	3.05∼13.65	Gamma	*
BSC per cycle	339	271.2∼406.8	Gamma	(Long et al., 2025)
Terminal care	2475	1,980∼2,970	Gamma	(Long et al., 2025)
Follow-up per cycle	240.19	192.15∼288.23	Gamma	(Wang et al., 2023)
Proteinuria per event	145.88	116.7∼175.06	Gamma	(S et al., 2018)
Decreased WBC count per event	186.57	149.26∼223.88	Gamma	(Long et al., 2024)
Anemia per event	5.53	4.42∼6.64	Gamma	(Long et al., 2024)
Decreased neutrophil count per event	16.62	13.3∼19.94	Gamma	(Long et al., 2024)
Cost (US$): USA				
Ivonescimab per 100 mg	802.35	534.9∼1,069.8	Gamma	​
Tislelizumab per 100 mg	5,452.58	4,362.06∼6,543.1	Gamma	*
Paclitaxel per 30 mg	9.81	7.85∼11.77	Gamma	*
Carboplatin per 50 mg	8.2	6.56∼9.84	Gamma	*
Docetaxel per 20 mg	17.52	14.02∼21.02	Gamma	*
BSC per cycle	3,728	2,982.4∼4,473.6	Gamma	(Tang et al., 2025)
Terminal care per cycle	14,532	11,625.6∼17,438.4	Gamma	(Tang et al., 2025)
Follow-up per cycle	2,374	1,899.2∼2,848.8	Gamma	(Tang et al., 2025)
Proteinuria per event	44.3	35.44∼53.16	Gamma	(Wei et al., 2024)
Decreased WBC count per event	13,105	10,484∼15,726	Gamma	(Shao et al., 2023)
Anemia per event	7,941	6,352.8∼9,529.2	Gamma	(Shao et al., 2023)
Decreased neutrophil count per event	13,656	10,924.8∼16,387.2	Gamma	(Shao et al., 2023)
Incidence of ADRs in the Ivonescimab group				
Proteinuria	5%	4%∼6%	Beta	(Lu et al., 2026)
Decreased WBC count	11%	8.8%∼13.2%	Beta	(Lu et al., 2026)
Anemia	7%	5.6%∼8.4%	Beta	(Lu et al., 2026)
Decreased neutrophil count	32%	25.6%∼38.4%	Beta	(Lu et al., 2026)
Incidence of ADRs in the Tislelizumab group				
Decreased WBC count	9%	7.2%∼10.8%	Beta	(Lu et al., 2026)
Anemia	5%	4%∼6%	Beta	(Lu et al., 2026)
Decreased neutrophil count	26%	20.8%∼31.2%	Beta	(Lu et al., 2026)
Utility value				
China: PFS	0.804	0.536∼0.84	Beta	(Nafees et al., 2017)
China: PD	0.321	0.05∼0.473	Beta	(Nafees et al., 2017)
USA: PFS	0.71	0.568∼0.852	Beta	(Chouaid et al., 2013)
USA: PD	0.67	0.536∼0.804	Beta	(Chouaid et al., 2013)
Disutility value				
Proteinuria	0.09	0.072∼0.108	Beta	(Zhou et al., 2025b)
Decreased WBC count	0.09	0.072∼0.108	Beta	(Zhu et al., 2022)
Anemia	0.073	0.058∼0.088	Beta	(Nafees et al., 2017)
Decreased neutrophil count	0.09	0.072∼0.108	Beta	(Zhu et al., 2022)
Proportion of patients receiving second-line treatment				
Ivonescimab group	36%	28.8%∼43.2%	Beta	(Lu et al., 2026)
Tislelizumab group	37%	29.6%∼44.4%	Beta	(Lu et al., 2026)
Others: China				
Discount rate	4.5%	0∼5%	-	(Wu Jing, 2025)
Weight	65	52∼78	Normal	(Luo et al., 2022; Zhou et al., 2025a)
Body surface area	1.72	1.376∼2.064	Normal	(Luo et al., 2022; Zhou et al., 2025a)
Creatinine clearance	70	56∼84	Normal	(Luo et al., 2022; Zhou et al., 2025a)
Others: USA				
Discount rate	3%	0∼7%	-	(Weinstein et al., 1996)
Weight	70	56∼84	Normal	(Luo et al., 2022; Zhou et al., 2025a)
Body surface area	1.79	1.43∼2.15	Normal	(Luo et al., 2022; Zhou et al., 2025a)
Creatinine clearance	73	58.4∼87.6	Normal	(Luo et al., 2022; Zhou et al., 2025a)

* Refers to The Menet (https://www.menet.com.cn/) or The Drug. price guide (https://www.drugs.com/price-guide/).

BSC: Best supportive care, WBC: White blood cell, ADRs: Adverse reactions, PFS: Progression-free survival, PD: Progressive disease.

The health utility value is a quantitative indicator of quality of life, with a utility value of 1 for complete health and 0 for death. The utility values of PFS (0.804) and PD (0.321) in Chinese NSCLC patients were obtained from the Chinese subgroup data of an international multicenter study based on the time trade-off method (Nafees et al., 2017). The data of U.S. NSCLC patients (PFS: 0.71, PD: 0.67) were derived from the results of a multi-center real-world study based on the EQ-5D tool (Chouaid et al., 2013). Disutility values associated with ADRs were also incorporated into the model, as detailed in Table 1.

Pharmacoeconomic methodology stipulates that both future costs and health outputs should be discounted when the time horizon exceeds 1 year. Referring to the China guidelines for pharmacoeconomic evaluations (2025), the annual discount rate was specified as 4.5% (0%–5%) (Wu Jing, 2025). According to the recommendations of the U.S. Public Health Service, the discount rate in the United States was established at 3% (0%–7%) (Weinstein et al., 1996).

### 2.5 Basic analysis

ICER was employed as the primary metric and compared against WTP to assess the cost-effectiveness of the ivonescimab regimen, with values below the WTP deemed cost-effective. Consistent with the recommendation of China guidelines for pharmacoeconomic evaluations (2025), the Chinese WTP was specified at double the national per-capita gross domestic product (GDP) for 2025, corresponding to $27,906/QALY (Wu Jing, 2025). In the U.S., the WTP was determined within a range of $100,000/QALY to $150,000/QALY (Eichler et al., 2004).

### 2.6 Sensitivity analysis

To assess parameter uncertainty, one-way sensitivity analysis (OWSA) and probabilistic sensitivity analysis (PSA) were conducted. OWSA evaluated the impact of individual parameter variation on the ICER. The drug cost boundaries came from the Menet database, whereas those without a specified source were assigned a ±20% variation around their base value. The findings were illustrated in a tornado diagram. PSA was performed using second-order Monte Carlo simulation (1,000 iterations), sampling from predefined parameter distributions to characterize the ICER distribution and estimate the probability of cost-effectiveness, thereby evaluating overall model uncertainty. In this study, cost parameters follow a gamma distribution, while ADR incidence rates and utility values conform to a beta distribution. A cost-effectiveness scatterplot and cost-effectiveness acceptability curve (CEAC) served as the primary visual tools for presenting the findings of PSA.

### 2.7 Scenario analysis

Scenario Analysis 1: This scenario analysis was conducted within the Chinese healthcare system, using the latest large-scale, multi-center health utility value study results from China (PFS: 0.809, PD: 0.776) to assess the impact of health utility values on the model (Wang et al., 2026).

Scenario Analysis 2: To assess the impact of subsequent treatment plans on the model, this study set up alternative second-line treatment scenarios. In China, according to the CSCO guidelines, gemcitabine (level II recommendation) was used instead of docetaxel (level I recommendation) (CSCO Oncology, 2026). In the U.S., according to the NCCN guidelines, docetaxel combined with ramucirumab was used instead of docetaxel monotherapy (Riely et al., 2026).

### 2.8 Exploratory analysis

To evaluate the uncertainty associated with the extrapolation of fitted survival curves, the following two alternative models were incorporated in this exploratory analysis (Bray et al., 2024). Exponential distribution: this distribution best matched the OS curve of the ivonescimab arm in the observation period based on the AIC and BIC (Wu et al., 2024). Mixture cure model (MCM): given the possibility of cure associated with immunotherapy, the MCM was applied to extrapolate the OS curves for both treatment groups.

### 2.9 Threshold analysis

To explore the theoretical cost-effective price threshold of ivonescimab, price threshold analysis was conducted. In the Chinese context, the retail price of ivonescimab was allowed to vary across a range of $0 to $150, with WTP thresholds defined as 1×, 2×, and 3× the Chinese per-capita GDP. For the U.S. market, the price range was set from $0 to $2,000, and WTP thresholds were applied at $100,000/QALY and $150,000/QALY.

## 3 Results

### 3.1 Results of basic analysis

Table 2 presents the findings from the basic analysis. In the Chinese setting, the ivonescimab group incurred an aggregate expenditure of $60,942 and yielded a health outcome of 2.33 QALYs, compared with $30,093 and 1.57 QALYs for the tislelizumab group, yielding an ICER of $40,328/QALY. In the U.S. setting, the costs for the two groups were $572,981 and $419,922, while the health outcomes were 3.16 QALYs and 2.19 QALYs, resulting in an ICER of $157,444/QALY.

**TABLE 2:** Results of basic analysis.

Group	Cost ($)	Incremental cost ($)	QALYs	Incremental QALYs	ICER ($/QALY)
China					
Tislelizumab group	30,093	​	1.57	​	​
Ivonescimab group	60,942	30,849	2.33	0.76	40,328
USA					
Tislelizumab group	419,922	​	2.19	​	​
Ivonescimab group	572,981	153,059	3.16	0.97	157,444

QALYs: Quality-adjusted life years, ICER: Incremental cost-effectiveness ratio.

### 3.2 Results of sensitivity analysis

Figure 1 depicts the results of the OWSA. In China, the parameters most strongly influencing the ICER were PD-stage utility, ivonescimab price, patient weight, and PFS-stage utility. In the United States, ivonescimab price was the dominant driver, followed by patient weight and tislelizumab price. Figures 2, 3 present the outcomes of PSA. In China, the probability of ivonescimab being cost-effective was 0.2% and 15.1% at WTP of 2× GDP ($27,906/QALY) and 3× GDP ($41,859/QALY), respectively, and exceeded 50% at $54,900/QALY. In the United States, this probability was 3.5% at $100,000/QALY, 44.2% at $150,000/QALY, and exceeded 50% at $157,900/QALY.

**FIGURE 1:**
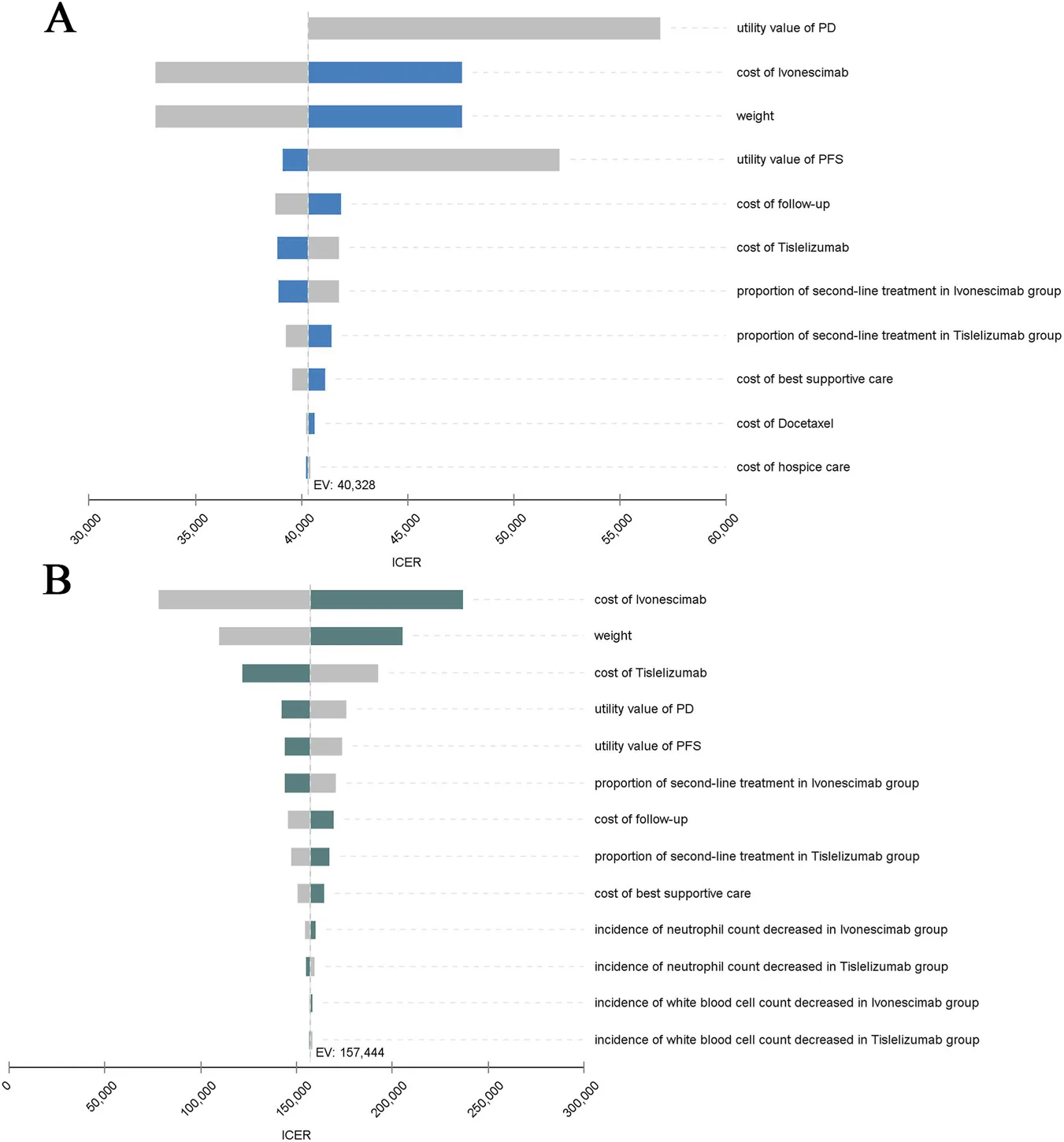
Tornado diagram. **(A)** Tornado Diagram in China, **(B)** Tornado Diagram in the United States, PFS: Progression-free survival, PD: Progressive disease, ICER: Incremental cost-effectiveness ratio, EV: Expected value.

**FIGURE 2:**
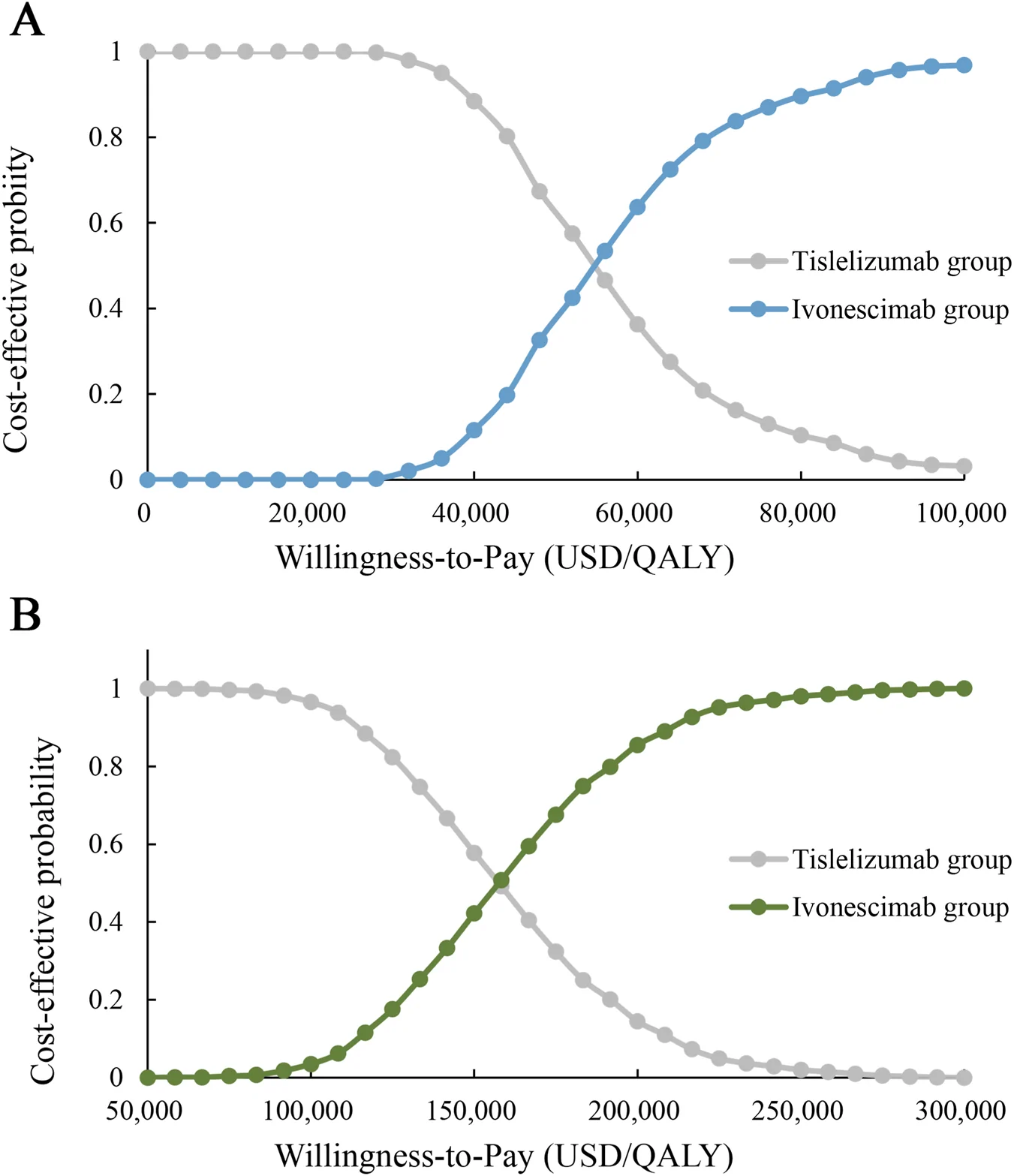
Cost‐effectiveness acceptability curve. **(A)** Cost‐effectiveness acceptability curve in China. **(B)** Cost‐effectiveness acceptability curve in the United States. WTP: willingness‐to‐pay, QALY:quality‐adjusted life‐year.

**FIGURE 3:**
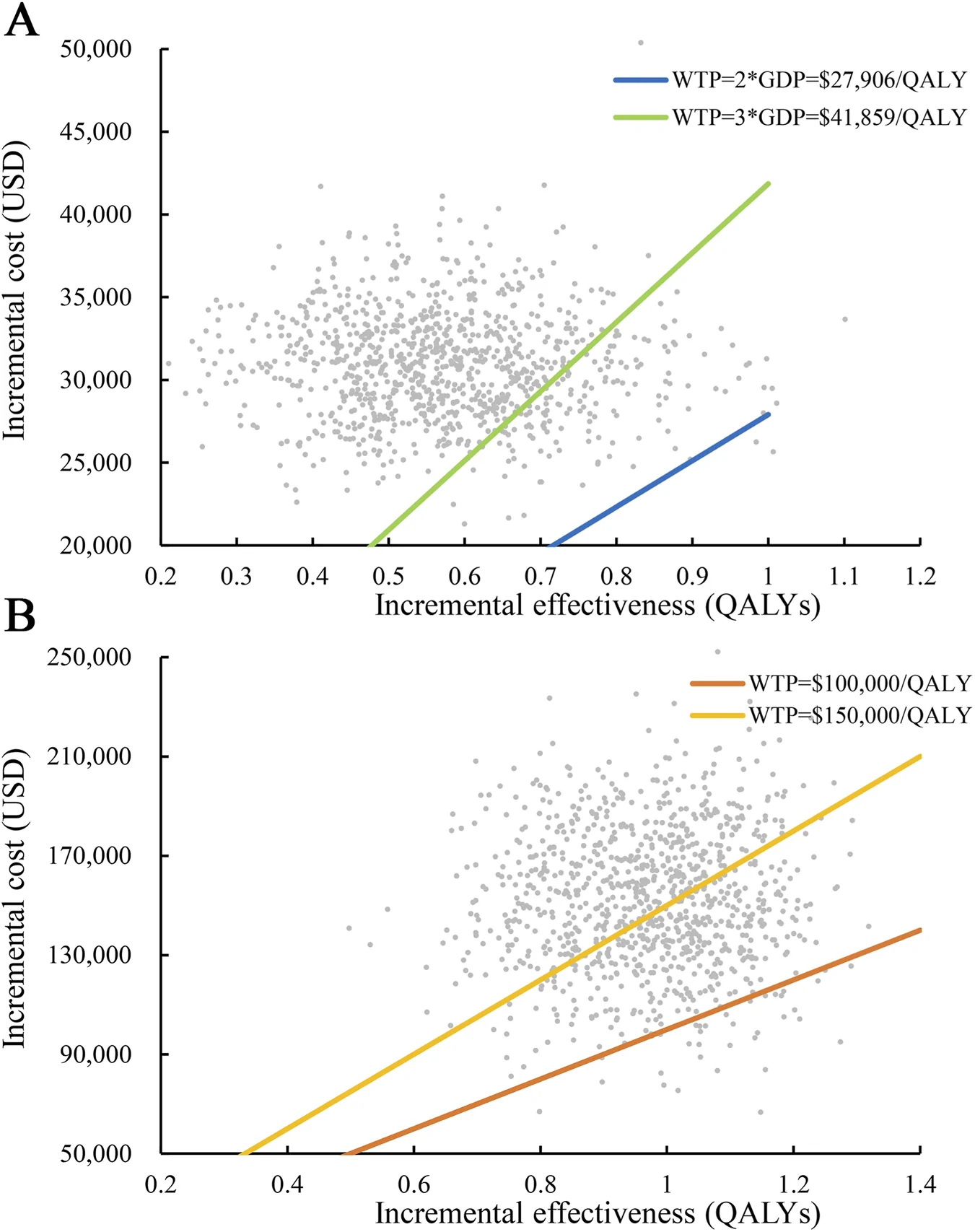
Cost‐effectiveness scatterplot. **(A)** Cost‐effectiveness scatterplot in China. **(B)** Cost‐effectiveness scatterplot in the United States. WTP: willingness‐to‐pay, QALY: quality‐adjusted life‐year.

### 3.3 Results of scenario analysis

Table 3 presents the outcomes from the scenario analysis.

**TABLE 3:** Results of scenario and exploratory analyses.

Group	Cost ($)	Incremental cost ($)	QALYs	Incremental QALYs	ICER ($/QALY)
Scenario analysis 1					
Tislelizumab group	30,093	​	2.52	​	​
Ivonescimab group	60,094	30,849	3.64	1.12	27,601
Scenario analysis 2: China					
Tislelizumab group	30,161	​	1.57	​	​
Ivonescimab group	61,032	30,872	2.33	0.76	40,358
Scenario analysis 2: the United States					
Tislelizumab group	420,041	​	2.19	​	​
Ivonescimab group	762,458	200,290	3.16	0.97	206,028
Exploratory analysis: Exponential distribution (China)					
Tislelizumab group	30,093	​	1.57	​	​
Ivonescimab group	56,219	26,127	2.13	0.57	46,010
Exploratory analysis: Mixture cure model (China)					
Tislelizumab group	30,104	​	1.57	​	​
Ivonescimab group	56,017	25,913	2.13	0.56	46,334
Exploratory analysis: Exponential distribution (the United States)					
Tislelizumab group	419,922	​	2.19	​	​
Ivonescimab group	523,447	103,525	2.75	0.56	184,546
Exploratory analysis: Mixture cure model (the United States)					
Tislelizumab group	420,041	​	2.19	​	​
Ivonescimab group	521,372	101,331	2.73	0.54	186,620

QALYs: Quality-adjusted life years, ICER: Incremental cost-effectiveness ratio.

Scenario Analysis 2: When the utility value changed, the health outcomes of the tislelizumab group and the ivonescimab group increased to 2.52 QALYs and 3.64 QALYs respectively, and the ICER decreased to $27,601/QALY.

Scenario Analysis 1: When the second-line treatment option changes, the ICER in China became $40,358/QALY, and the ICER in the U.S. became $206,028/QALY.

### 3.4 Results of exploratory analysis

For China, extrapolating the ivonescimab OS curve with the exponential distribution resulted in total costs estimated at $56,219, health output of 2.13 QALYs, and an ICER of $46,010/QALY. For the U.S, the corresponding cost was $523,447, health outcome was 2.75 QALYs, and the ICER increased to $184,546/QALY. When MCM was applied, the ICER in China became $46,334/QALY, and the ICER in the United States became $186,620/QALY.

### 3.5 Results of threshold analysis

Figure 4 displays the results of threshold analysis. In China, when the WTP thresholds were set at 1× GDP, 2× GDP, and 3× GDP, the theoretical cost-effective price threshold for ivonescimab were $27.85/100mg, $67.63/100mg, and $107.4/100mg, respectively. In the United States, under WTP of $100,000/QALY and $150,000/QALY, the corresponding theoretical cost-effective price threshold were $609.1/100 mg and $777.31/100mg, respectively.

**FIGURE 4:**
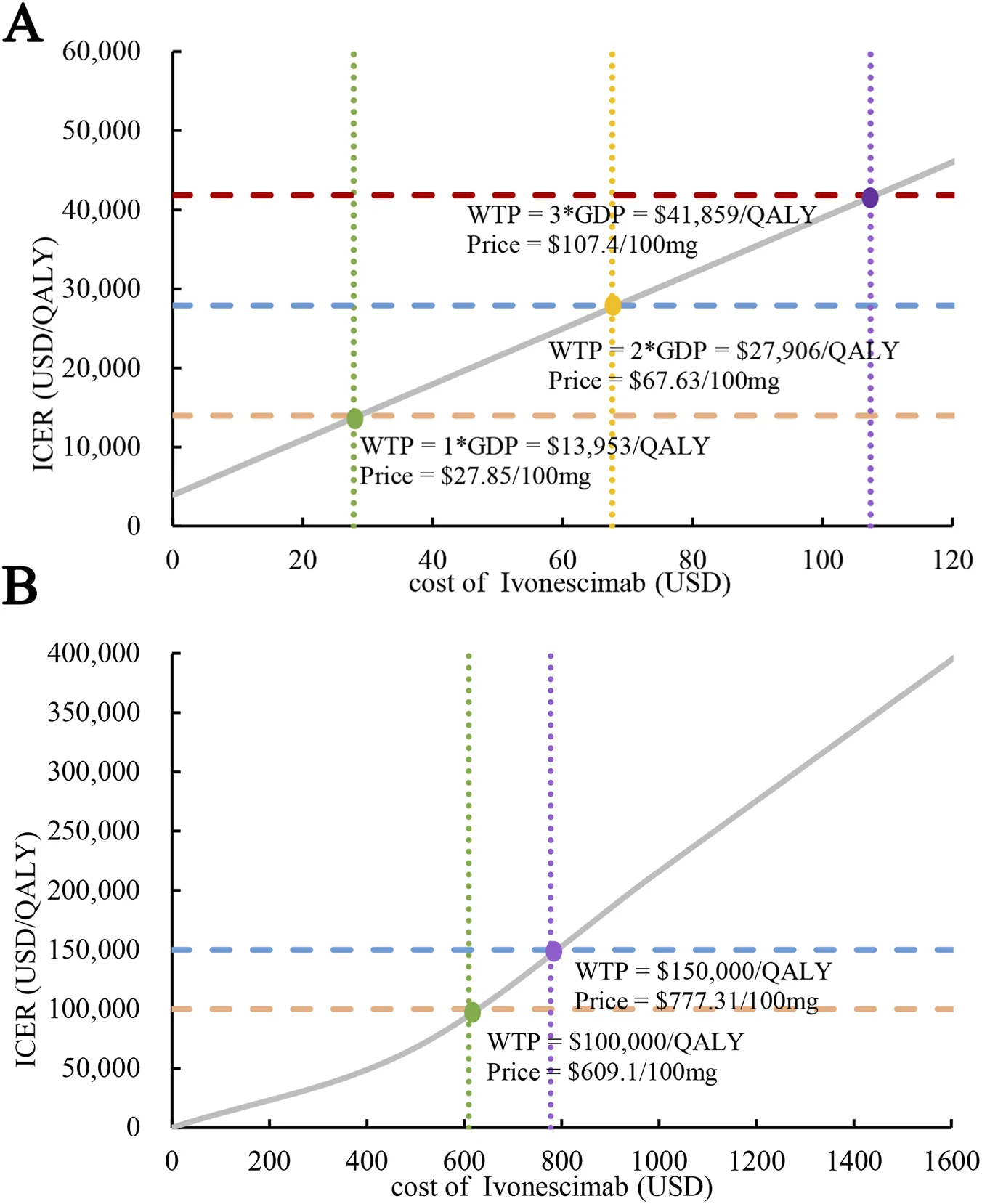
Threshold Analysis. **(A)** Threshold Analysis in China, **(B)** Threshold Analysis in the United States, WTP: Willingness-to-pay, QALY: Quality-adjusted life years.

## 4 Discussion

This is the first study to systematically appraise the cost-effectiveness of ivonescimab plus chemotherapy as an initial treatment for advanced squamous NSCLC, framed within the healthcare system contexts of both China and the United States. The results showed that although the ivonescimab regimen was not cost-effective in either country, there was a significant difference in the probability of its cost-effectiveness advantage. Under the unified model framework of this study, this difference is the result of the combined effect of multiple factors. Firstly, the pricing mechanisms for drugs in the two countries are different. In China, drugs enter the market at low prices through national medical insurance negotiations, while in the United States, drug pricing is highly market-driven. Secondly, there is a significant difference in health utility values between the two countries. The utility value of the PD stage for Chinese patients is much lower than that of American patients (China: 0.321, U.S.: 0.67), and the utility value, in turn, has a significant impact on ICER through its influence on QALY. Thirdly, the discount rate (China: 4.5%, U.S.: 3%) has differential impacts on the discounting of cumulative QALY and costs in the long-term survival extrapolation. Finally, there are also marked differences in the WTP between the two countries, which determines the criteria for evaluating the cost-effectiveness acceptability. However, it should be noted that there are systematic differences in medical service models, subsequent treatment regimens, and medical insurance reimbursement policies between the two countries. The unified model framework of this study did not take into account these heterogeneity factors.

The results of the OWSA revealed that, in the Chinese setting, the PD-state utility value exerted the strongest influence on the ICER. A plausible explanation is that patients occupy the PD state for a comparatively long portion of their remaining lifetime, coupled with the extremely low baseline utility value for the PD state (0.321). As a result, relatively small fluctuations in this utility value, when accumulated over a long-term horizon, translate into marked changes in total QALY estimates. In the U.S., the ICER responded most sharply to costs of ivonescimab and tislelizumab, as well as to patient body weight. This is likely because the incremental costs ($153,059) predominantly accrued during the PFS phase ($89,934), and the cost difference between ivonescimab and tislelizumab drove these incremental costs. Furthermore, given that the dosage of ivonescimab is linearly correlated with body weight (20 mg/kg), all three factors substantially affected the ICER. This difference indicates that in China, drug prices are lower, and the ICER is more sensitive to the marginal changes in health outcomes (QALYs). The significant improvement in patients’ quality of life significantly affects the results of drug economic evaluation. In the United States, drug prices are higher, and the ICER is more sensitive to the marginal changes in cost parameters. The results of drug economic evaluation are more dependent on the price of drugs themselves.

The results of PSA showed that the economics of the ivonescimab regimen were highly sensitive to the WTP. In the Chinese setting, the cost-effectiveness acceptability of the ivonescimab group reached merely 0.2% at a WTP of 2× GDP *per capita*. It was not until the WTP was raised to $54,900/QALY that the cost-effectiveness results became relatively stable. With future economic growth and the advancement of policies such as national health insurance negotiations and volume-based procurement, the cost-effectiveness of the ivonescimab regimen is expected to further improve. In the United States, the probability of cost-effectiveness was 3.5% at $100,000/QALY and 44.2% at $150,000/QALY, exceeding 50% only at $157,900/QALY. This indicates that the price of ivonescimab in the United States has exceeded the mainstream willingness to pay, and price reduction is a key measure to enhance its economic viability.

In scenario analysis 2, substituting gemcitabine for docetaxel in China yielded an ICER of $40,358/QALY, comparable to the basic case ($40,328/QALY). In the United States, adding ramucirumab to docetaxel increased the ICER to $206,028/QALY, exceeding the basic analysis ($157,444/QALY). The impact of second-line treatment on ICER depends on the difference in the proportion of second-line treatments between the two groups and the cost difference of the treatment plans themselves. In the HARMONi-6 trial, the proportions of second-line treatments were similar (37% vs. 36%), so the cost difference was mainly determined by the cost of the treatment plans. The cycle costs of gemcitabine and docetaxel in China were similar (gemcitabine: $41.27, docetaxel: $36.19), so the ICER remained largely unchanged. In the United States, the cycle cost of the combined treatment plan was much higher than that of the single drug ($10,744.9 vs. $117.6), thus the ICER increased significantly. After the change in the second-line treatment plan, the ICER remained significantly higher than the WTP, indicating that the model has good robustness in terms of the second-line treatment assumption.

Both the log-logistic and log-normal distributions display a hazard trajectory that is non-monotonic, characterized by an initial rise followed by a subsequent decline over time. This pattern aligns with the clinical characteristics of immunotherapy, namely early immune activation followed by the establishment of immunological memory in the later phase. Consequently, these distributions demonstrate robust performance in predicting long-term survival, particularly when the survival data are not yet mature (Monnickendam, 2024). Guided by AIC and BIC, the log-logistic and log-normal distributions were selected to fit and extrapolate OS curves in the basic analysis. To assess the impact of model selection on the ICER, two alternative models were included in the exploratory analysis (Bray et al., 2024). Exponential distribution: Although this distribution achieved the smallest AIC and BIC values for the observed OS curve of the ivonescimab group, its assumption of a constant hazard conflicts with the potential mechanism of immunotherapy, which may yield a long-term survival plateau. Therefore, this distribution is recommended only as a conservative reference for interpretation (Wu et al., 2024). MCM: This model assumes that a subset of patients achieves long-term cure following immunotherapy. It is therefore applicable in therapeutic areas where a proportion of patients may be considered cured, and the survival curve exhibits a prolonged tailing pattern. The MCM has also been adopted occasionally in the context of tumor immunotherapy (Felizzi et al., 2021). However, MCM requires mature survival data as support. Applying it to immature early data may lead to an overestimation of survival periods. The survival data from the HARMONi-6 trial are not yet mature, so its results likewise warrant cautious interpretation.

Threshold Analysis is a theoretical value derived from the model framework and parameter assumptions of this study. Since a series of parameters, such as survival extrapolation models, health utility values, and medical costs, significantly affect the ICER, the price threshold will also fluctuate accordingly. Drug market pricing is a complex decision-making process involving multiple stakeholders, but we have not considered these real-world factors. Therefore, the price threshold in this study serves only as a theoretical exploration to reveal the cost-effectiveness boundary of ivonescimab under current evidence conditions.

This study also has some limitations. Firstly, the U.S. analysis is based on the hypothetical price of ivonescimab. Therefore, the U.S. analysis should be understood as a scenario exploration for pricing, rather than a confirmatory assessment of cost-effectiveness. Secondly, this study extrapolates the efficacy data from the HARMONi-6 trial (based on a Chinese population) to the U.S. population and uses parametric models to extend survival curves beyond the observation period, both of which may introduce certain biases. Thirdly, since the HARMONi-6 trial did not publish any quality-of-life data, the utility value data were obtained from other studies. Fourthly, unifying and simplifying drug treatment regimens for PD stages may affect drug treatment costs. Finally, the cost of ADR treatment only takes into account grade 3–4 ADRs with an incidence above 5%. Although this is a common practice in the economic evaluation of oncology drugs, this simplification may lead to an underestimation of the total cost of ADRs.

## 5 Conclusion

This study evaluated the cost-effectiveness of first-line ivonescimab plus chemotherapy versus tislelizumab plus chemotherapy for advanced squamous NSCLC from Chinese and U.S. healthcare perspectives, and estimated theoretical cost-effective price threshold of ivonescimab. In China, at a WTP of 2× GDP *per capita*, the ivonescimab regimen was not cost-effective. When the WTP were set at 1×, 2×, and 3× GDP *per capita*, the theoretical cost-effective price threshold for ivonescimab were $27.85/100mg, $67.63/100mg, and $107.4/100 mg. In the U.S., the ivonescimab strategy was also not cost-effectiv even at the WTP of $150,000/QALY. Under WTP of $100,000/QALY and $150,000/QALY, the theoretical cost-effective price threshold of ivonescimab were $609.1/100 mg and $777.31/100mg, respectively. These findings provide exploratory evidence for value-based pricing discussions. However, all price thresholds are conditional on model assumptions and selected WTP values, should not be interpreted as pricing recommendations, and real-world decisions require additional considerations including budget impact, payer negotiations, and local practice.

## Data Availability

The original contributions presented in the study are included in the article/Supplementary Material, further inquiries can be directed to the corresponding authors.
