# Supplementary material for: Cost-effectiveness analysis of ivonescimab plus chemotherapy in advanced squamous NSCLC: a Chinese healthcare system perspective with U.S. pricing scenario exploration
Source: Front Pharmacol. 2026 Oct 27;17:1942976. doi: 10.3389/fphar.2026.1942976 (PMC13747759; doi:10.3389/fphar.2026.1942976)
Supplement: Supplementary file 1 [file DataSheet1.docx]

Supplementary Material

# 1 Supplementary Figures and Tables

## 1.1 Supplementary Figures

**Supplementary Figure 1.** Schematic of the model structure. NSCLC: non‑Small cell lung cancer, PFS: Progression-free survival, PD: Progressive disease.

**Supplementary Figure 2.** **Kaplan-Meier Curve Fitting and Extrapolation.** (A):

Survival curves in the Ivonescimab group, (B): PFS: Survival curves in the Tislelizumab group, Progression-free disease, OS: Overall survival, K-M: Kaplan-Meier, MCM: Mixture cure model

## 1.2 Supplementary Tables

**Table S1.** CHEERS checklist 2022

| **Topic** | **No** | **Item** | **Reported** |
| --- | --- | --- | --- |
| **Title** | | | |
| Title | 1 | Identify the study as an economic evaluation and specify the interventions being compared | Yes |
| **Abstract** | | | |
| Abstract | 2 | Provide a structured summary that highlights context, key methods, results, and alternative analyses | Yes |
| **Introduction** | | | |
| Background and objectives | 3 | Give the context for the study, the study question, and its practical relevance for decision making in policy or practice | Yes |
| **Methods** | | | |
| Health economic analysis plan | 4 | Indicate whether a health economic analysis plan was developed and where available | Not applicable |
| Study population | 5 | Describe characteristics of the study population (such as age range, demographics, socioeconomic, or clinical characteristics) | Yes |
| Setting and location | 6 | Provide relevant contextual information that may influence findings | Yes |
| Comparators | 7 | Describe the interventions or strategies being compared and why chosen | Yes |
| Perspective | 8 | State the perspective(s) adopted by the study and why chosen | Yes |
| Time horizon | 9 | State the time horizon for the study and why appropriate | Yes |
| Discount rate | 10 | Report the discount rate(s) and reason chosen | Yes |
| Selection of outcomes | 11 | Describe what outcomes were used as the measure(s) of benefit(s) and harm(s) | Yes |
| Measurement of outcomes | 12 | Describe how outcomes used to capture benefit(s) and harm(s) were measured | Yes |
| Valuation of outcomes | 13 | Describe the population and methods used to measure and value outcomes | Yes |
| Measurement and valuation of resources and costs | 14 | Describe how costs were valued | Yes |
| Currency, price date, and conversion | 15 | Report the dates of the estimated resource quantities and unit costs, plus the currency and year of conversion | Yes |
| Rationale and description of model | 16 | If modelling is used, describe in detail and why used. Report if the model is publicly available and where it can be accessed | Yes |
| Analytics and assumptions | 17 | Describe any methods for analyzing or statistically transforming data, any extrapolation methods, and approaches for validating any model used | Yes |
| Characterizing heterogeneity | 18 | Describe any methods used for estimating how the results of the study vary for subgroups | Not applicable |
| Characterizing distributional effects | 19 | Describe how impacts are distributed across different individuals or adjustments made to reflect priority populations | Not applicable |
| Characterizing uncertainty | 20 | Describe methods to characterize any sources of uncertainty in the analysis | Yes |
| Approach to engagement with  patients and others affected by the  study | 21 | Describe any approaches to engage patients or service recipients, the general public, communities, or stakeholders (such as clinicians or payers) in the design of the study | Not applicable |
| **Results** | | | |
| Study parameters | 22 | Report all analytic inputs (such as values, ranges, references) including uncertainty or distributional assumptions | Yes |
| Summary of main results | 23 | Report the mean values for the main categories of costs and outcomes of interest and summarise them in the most appropriate overall measure | Yes |
| Effect of uncertainty | 24 | Describe how uncertainty about analytic judgments, inputs, or projections affect findings. Report the effect of choice of discount rate and time horizon, if applicable | Yes |
| Effect of engagement with patients and others affected by the study | 25 | Report on any difference patient/service recipient, general public, community, or stakeholder involvement made to the approach or findings of the study | Not applicable |
| **Discussion** | | | |
| Study findings, limitations, generalizability, and current knowledge | 26 | Report key findings, limitations, ethical or equity considerations not captured, and how these could affect patients, policy, or practice | Yes |
| Other relevant information Source of funding | 27 | Describe how the study was funded and any role of the funder in the identification, design, conduct, and reporting of the analysis | Yes |
| Conflicts of interest | 28 | Report authors conflicts of interest according to journal or International Committee of Medical Journal Editors requirements | Yes |

**Table S2.** Comparison analysis results of survival time

| Variable | Ivonescimab group | Tislelizumab group |
| --- | --- | --- |
| Original mPFS (95%CI) | 11.1 months (9.9-NE) | 6.9 months (5.8-8.6) |
| Fitted mPFS (95%CI) | 11.2 months (9.91-NE) | 6.92 months (5.9-8.55) |
| Original mOS (95%CI) | 27.9 months (27.89-NE) | 23.7 months (20.11-NE) |
| Fitted mOS (95%CI) | 27.9 months (27.9-NE) | 23.6 months (20.5-NE) |

mPFS: median Progression-free survival, OS: median Overall survival, NE: Not evaluable

**Table S3.** Goodness-of-fit results

| Variable | Exponential | Weibull | Gompertz | Log-logistic | Log-normal | Gamma |
| --- | --- | --- | --- | --- | --- | --- |
| PFS of the Ivonescimab group | | | | | | |
| AIC | 741.0777 | 729.8355 | 734.4499 | 729.6991 | 735.5884 | 729.7588 |
| BIC | 744.6612 | 737.0035 | 741.6169 | 736.8661 | 742.7554 | 736.9258 |
| PFS of the Tislelizumab group | | | | | | |
| AIC | 903.8244 | 887.2683 | 901.9211 | 873.7491 | 879.2703 | 882.683 |
| BIC | 904.4079 | 894.4354 | 909.0881 | 880.9161 | 886.4373 | 889.85 |
| OS of the Ivonescimab group | | | | | | |
| AIC | 843.4262 | 845.3951 | 845.3775 | 845.9406 | 849.6816 | 845.4002 |
| BIC | 847.0097 | 852.5621 | 852.5445 | 853.1076 | 856.8486 | 852.5672 |
| OS of the Tislelizumab group | | | | | | |
| AIC | 1106.553 | 1097.161 | 1102.566 | 1095.475 | 1094.556 | 1095.945 |
| BIC | 1110.137 | 1104.328 | 1109.733 | 1102.642 | 1101.723 | 1103.112 |

PFS: Progression-free survival, OS: Overall survival, AIC: Akaike Information Criterion, BIC: Bayesian Information Criterion.

**Table S4**. Fitted parameter

| Variable | Optimal distribution function | Baseline value |
| --- | --- | --- |
| PFS curve | | |
| Ivonescimab group | Log-logistic | shape: 1.63235  scale: 12.23573 |
| Tislelizumab group | Log-logistic | shape: 1.8356  scale: 7.90506 |
| OS curve | | |
| Ivonescimab group | Log-logistic | shape: 1.12008  scale: 39.26095 |
| Tislelizumab group | Log-normal | meanlog: 3.16125  sdlog: 1.14611 |
| PFS: Progression-free survival, OS: Overall survival | | |
